# Nongovernmental Organization Practitioners’ Perspectives on the Challenges and Solutions to Changing Handwashing Behavior in Older Children: A Qualitative Study

**DOI:** 10.9745/GHSP-D-22-00231

**Published:** 2023-02-28

**Authors:** Julie Watson, Oliver Cumming, Robert Dreibelbis

**Affiliations:** a Department of Disease Control, London School of Hygiene and Tropical Medicine, London, United Kingdom.

## Abstract

**Background:**

Increasing handwashing with soap (HWWS) among older children (aged 5–14 years) can achieve a substantial public health impact. However, HWWS interventions targeting older children have had mixed success. Recent research has attempted to quantitatively identify effective intervention techniques; however, success is likely also influenced by the wider context of implementation. We explore nongovernmental organization (NGO) practitioners’ perspectives on the challenges and solutions to HWWS interventions targeting older children to enhance understanding of what is required, beyond intervention content, for them to be effective.

**Methods:**

We conducted in-depth, semistructured interviews in April–November 2020 with 25 practitioners employed across 11 NGOs and involved in HWWS interventions targeting older children in development and humanitarian settings. We used purposive and snowball sampling to recruit participants in roles at the global, national/regional, and local levels. Interviews were audio-recorded, transcribed, and thematically analyzed to identify challenges and solutions to HWWS interventions targeting older children. Results were organized according to program development cycle stages.

**Results:**

Twelve themes relating to perceived challenges emerged: (1) lack of prioritization, (2) funding inconsistency, (3) insufficient formative research, (4) demand on resources, (5) unengaging intervention content, (6) non-enabling physical environments, (7) availability of skilled implementers, (8) reaching out-of-school children, (9) community mistrust, (10) lack of coordination, (11) lack of evaluation rigor, and (12) failure to assign older children’s HWWS as a primary outcome in evaluations of hygiene interventions. Recommended solutions were at the intervention, organization, and sector levels.

**Conclusion:**

Intervention design and delivery are important for the success of HWWS interventions for older children, but contextual factors, such as the availability of human and material resources and the level of coordination within and beyond the NGO sector, should also be considered. NGOs need to prioritize HWWS promotion among older children and support programs accordingly.

## Abstract (precis)

We explore the challenges to handwashing interventions targeting older children and recommended solutions from nongovernmental organization practitioners’ perspectives involved in the design, coordination, implementation, or evaluation of child-targeted HWWS interventions.

## Teaser

**Key Findings:**

- Improving handwashing with soap (HWWS) practice among older children can reduce disease transmission, but funding for these interventions targeting older children is insufficient and inconsistent.
- Practitioners report that most HWWS interventions targeting older children are resource-heavy, unengaging, and implemented within non-enabling physical environments, with little evidence of success.
- Lack of coordination within and between sectors creates major challenges to implementing HWWS interventions targeting children.

**Key Implications:**

- The nongovernmental organization (NGO) sector should prioritize improving older children’s handwashing and support programs accordingly.
- NGO practitioners should balance resource needs against using evidence-based, engaging approaches that address the drivers of handwashing among older children.
- NGOs should foster better intra- and intersectoral coordination to overcome challenges related to the integration of HWWS promotion within existing educational institutions, the standardization of implementation tools, and the sustainability of interventions.

## INTRODUCTION

Handwashing with soap (HWWS) at critical times is one of the most effective ways to reduce the spread of infectious diseases. Meta-analyses suggest that HWWS can lead to reductions in diarrheal disease by 23%–43%^1^^–^^3^ and acute respiratory infections by 21%–23%.^4^^,^^5^ HWWS has also been associated with reductions in neglected tropical diseases such as trachoma^6^ and some soil-transmitted helminth infections.^7^

Although the largest burden is borne by children aged younger than 5 years, diarrhea and acute respiratory infections account for more than 14% of mortality among children aged 5–14 years and are the third and fourth leading causes of death in this age group, respectively.^8^ Improving HWWS among older children has significant public health potential. Not only does HWWS reduce disease transmission, but it is also associated with reduced rates of school absenteeism,^9^^–^^13^ which may lead to improved academic attainment^14^^,^^15^ and associated economic and health benefits later in life.^16^ Keeping children in school is also important for access to vaccination and nutrition programs, access to mental health and psychosocial support services, and child protection risks.^17^^–^^19^

The responsibility for younger children’s HWWS usually falls to the caregiver; thus, interventions aimed at reducing infectious disease transmission among young children are typically targeted at the caregiver level. However, older children are more independent; they may be about to enter or are already in school and have more agency over the cleanliness of their own hands. Habits are formed during older childhood that may persist into adulthood.^20^^–^^22^ Older children may also act as agents of change among their peers, encouraging others to practice HWWS.^23^^–^^28^

Wide-scale HWWS programs targeting older children are typically a major component of an outbreak response, specifically, school-based programs designed to improve handwashing behaviors to prevent the spread of respiratory and gastrointestinal infections between students and the broader community.^10^^,^^11^^,^^29^^–^^31^ Recently, programs aimed at encouraging older children to perform regular HWWS in school have been central to safe school-reopening strategies and for preventing school closures.^32^

Despite the clear need for effective interventions targeting HWWS practices among older children, there is a dearth of evidence on how best to design and deliver these interventions. HWWS interventions targeting older children are predominantly school-based or focused on child-friendly spaces (CFS)—safe places set up in an emergency-affected community that help children return to a normal routine by offering activities, games, and informal education—and are implemented by teachers or hygiene promoters.^33^^–^^35^ Health education and health-related knowledge transfer largely drive these interventions and have had mixed success.^34^^–^^36^ In a recent systematic review, some specific intervention techniques that may be effective in increasing older children’s hand hygiene were identified; however, this review did not consider the wider context of implementation, which may influence effectiveness.^34^

Despite the clear need for effective interventions targeting HWWS practices among older children, there is a dearth of evidence on how best to design and deliver these interventions.

Nongovernmental organizations (NGOs) are often engaged in HWWS programs in development and humanitarian emergency settings.^33^ Therefore, NGO practitioners can offer invaluable insights into the challenges facing HWWS interventions targeting older children and make pragmatic recommendations to improve effectiveness. Prior research exploring NGO practitioners’ perspectives on challenges to hygiene promotion has been limited to a humanitarian context and has not focused on HWWS interventions targeting older children.^37^^,^^38^ In this study, we qualitatively explore the challenges to HWWS interventions targeting older children and recommended solutions from the perspective of NGO practitioners involved in the design, coordination, implementation, or evaluation of child-targeted HWWS interventions.

## METHODS

### Study Design

A qualitative study involving in-depth, semistructured interviews followed by inductive thematic analysis was undertaken to provide a nuanced and detailed account of participants’ perceptions of challenges to HWWS interventions for older children and recommended solutions to these challenges.

### Participants and Sample Selection

Participants were NGO practitioners involved in designing, coordinating, implementing, and/or evaluating HWWS interventions targeting older children in development and humanitarian settings. This was the only eligibility criterion.

Purposive sampling was used to recruit information-rich participants via several channels. First, we contacted eligible individuals within our existing network to seek their consent to participate (n=6). Additional participants were recruited through the Global WASH Cluster—a global network of humanitarian water, sanitation, and hygiene (WASH) agencies—via an advertisement inviting interested network members to participate. Individuals who made contact were assessed for eligibility (n=11), and those eligible were invited to participate (n=8). Additional participants were recruited via referral from those already enrolled in the study (n=11). All communications before interviews were via email.

To capture a broad range of views and achieve theoretical saturation, we aimed to recruit a minimum of 18 practitioners employed across 6 or more NGOs, with at least 6 practitioners working at a global level, 6 at a regional or national level, and 6 at a local level. This initial target sample size of 18 was guided by previous similar research,^39^^–^^43^ but we continued recruitment until data reached theoretical saturation.

### Data Collection

In-depth, semistructured interviews were undertaken remotely via the Zoom virtual meeting platform between April and November 2020. All interviews were conducted in English by the lead author (JW)—a female academic researcher experienced in conducting and analyzing qualitative research. Interviews lasted between 45 and 90 minutes and were audio-recorded using the Zoom recording function. An interview guide that the authors developed and piloted facilitated the discussion on the challenges and solutions to HWWS interventions for older children but did not mandate rigid adherence to the questions or the order in which they appeared in the guide. Participants were encouraged to consider the determinants of older children’s handwashing behavior, discuss approaches to HWWS promotion they had experienced in the past, and give recommendations for future interventions. The full interview guide is available in Supplement 1.

### Data Management and Analysis

Audio recordings were transcribed verbatim, and transcripts were imported into QSR Nvivo 12 for analysis. The lead author conducted a thematic analysis of the transcripts following the 6-stage approach suggested by Braun and Clarke.^44^ This approach entails (1) becoming familiar with the data, (2) generating initial codes, (3) searching for themes, (4) reviewing themes, (5) defining themes, and (6) writing up. An inductive approach to coding was adopted to avoid making preliminary assumptions and to allow themes to emerge from the data. Emergent themes on challenges and related solutions were then organized along 4 key stages of the program development cycle: (1) funding acquisition, (2) design, (3) delivery, and (4) evaluation.^45^ The coding structure is available in Supplement 2. Direct representative quotations of the participant’s opinions are presented to support our analysis; however, to protect participants’ identities, we only attribute the quotation to the participant’s gender and the level of their role in the organization (global, regional/national, or local).

### Ethical Approval

Ethical approval for this study was granted by the London School of Hygiene and Tropical Medicine Ethics Review Committee (ref. 14483) before contacting participants. Participants were provided with a Participant Information Sheet detailing the study before giving their written consent via email and subsequent verbal consent at the start of the interview.

## RESULTS

A total of 25 participants were interviewed from across 11 different NGOs operating in both development and humanitarian settings. Ten of these were international NGOs and 1 was a national NGO. Of the participants interviewed, 36% worked at a global level (n=9), 24% worked at a regional or national level (n=6), and 40% worked at a local level (n=10) (Table 1). To maintain participants’ anonymity, we omitted details of their affiliated NGOs from the table.

**TABLE 1.** Characteristics of Participants in Interviews on Perceived Challenges to HWWS Interventions Targeting Older Children

**Position (N=25)**	**Gender**	**Current Professional Location **
Global level		
Global WASH Advisor	F	Belgium
Senior Behavior Change Advisor	F	Canada
Global WASH Advisor	F	India
Health Promotion and Community Engagement Advisor	F	Netherlands
Global Public Health Promotion Advisor	F	United Kingdom
Senior WASH Advisor	M	United Kingdom
WASH Consultant	F	United Kingdom
WASH Technical Advisor	F	United Kingdom
Senior WASH Advisor	M	United States
National level		
Hygiene Promoter	F	Lebanon
WASH Technical Working Group Head	M	Myanmar
Health Promotion and Community Engagement Specialist	M	Pakistan
National WASH Advisor	M	Philippines
Public Health Team Leader	M	Tanzania
Regional level		
WASH Regional Advisor	M	Lebanon
Local level		
Senior Innovation Officer (Public Health Promotion)	F	Bangladesh
Public Health Promotion Officer	M	Bangladesh
Organization Founder and Director	M	Cameroon
Senior Behavior Change Advisor	F	Madagascar
WASH Program Officer	F	Madagascar
Hygiene Promoter	F	Nigeria
Hygiene Promoter Manager	M	Nigeria
Hygiene Promoter Supervisor	M	Pakistan
Program Officer for School and Nutrition	F	Philippines
WASH Project Manager	M	Uganda

Abbreviations: HWWS, handwashing with soap; WASH, water, sanitation, and hygiene.

Themes describing the perceived challenges to HWWS interventions targeting older children in development and humanitarian contexts and related solutions are organized into the 4 stages of the program cycle: (1) funding acquisition, (2) design, (3) delivery, and (4) evaluation. The 12 themes that emerged across these stages interacted with and influenced one another in various ways (Table 2). We elaborate on the relationships between themes in the discussion.

**TABLE 2.** Summary of Emergent Themes From Participant Interviews on Perceived Challenges to Handwashing With Soap Interventions Targeting Older Children

**Program Cycle Stage**	**Theme**
Funding acquisition	Lack of prioritization
	Funding inconsistency
Design	Insufficient formative research
	Demand on resources
	Unengaging intervention content
	Non-enabling physical environments
Delivery	Availability of skilled implementers
	Reaching out-of-school children
	Community mistrust
	Lack of coordination
Evaluation	Lack of evaluation rigor
	Failure to assign older children’s handwashing as a primary outcome in evaluations of hygiene interventions

### Stage 1: Funding Acquisition

The theme of lack of prioritization emerged predominantly from interviews with global-level participants, whereas funding inconsistency was reflected across all levels of participants.

#### Lack of Prioritization

Participants reported that HWWS interventions targeting older children do not receive sufficient funding, reflecting a low prioritization of older children’s HWWS within the NGO sector. Participants believed that with numerous competing priorities, particularly in humanitarian settings, HWWS promotion is often deprioritized. Participants called for the sector to recognize the potential to achieve a large public health impact by targeting HWWS interventions to older children and to design more HWWS interventions for this specific age group.

*It does always feel a little bit like working with children specifically and understanding their specific needs is something that is a bit like “well that can come later,” it's not something to focus on at the beginning… If it's not in the budget then, not only do we then not have the resources to be able to do it, but I think sometimes in the busyness of an initial response it can quite often get forgotten because it's not listed down as a deliverable in the budget or in the proposal*. —Woman, global level

Participants reported that HWWS interventions targeting older children do not receive sufficient funding.

#### Funding Inconsistency

Where HWWS interventions targeting older children are funded, participants felt that funding changes throughout the program cycle (cuts in budget and short-term funding) make sustaining interventions difficult as they often result in discontinuity in program ownership.

*What we learned is that sustainability is very important because, unfortunately, not long afterwards it was the end of the (financial) year and [organization redacted] was not selected to continue as WASH lead in these camps. We are currently operational partner but not WASH lead and what that means is … we are no longer able to do these competitions in schools, we have to go through another partner and that brings a bit of complications.* —Man, national level

### Stage 2: Design

Four major themes related to intervention design emerged: insufficient formative research, demand on resources, unengaging intervention content, and non-enabling physical environments. Insufficient formative research emerged only from global- and regional-level participants. The other 3 themes were reflected across all participant levels.

#### Insufficient Formative Research

Participants reported that formative research is rarely undertaken to inform HWWS interventions targeting older children. Many recommended consultations with children to ensure interventions are appealing, appropriate, and acceptable and to allow messages to be customized to settings. Involving children in the design of HWWS interventions was considered a good way for them to engage and take more ownership of associated activities and handwashing facilities.

*I urge none of the organizations to design hygiene behavior anecdotally… it has to be more evidence based, it has to be informed based on formative research, the package has to be designed through a creative process so that at least the package is attractive, engaging, emotional to the school students.* —Man, global level

#### Demand on Resources

Existing HWWS interventions targeting older children were perceived to be too resource intensive to be very effective in resource-poor settings. Interventions were said to require numerous props, materials, and supplies that are not always affordable. They also often require health messages to be delivered consistently, which relies on highly skilled and motivated implementers with skillsets that are often difficult to find at the local level. Integrating intensive HWWS interventions into the school or CFS curricula was also perceived to be challenging as they pose a demand on teachers and CFS managers. Yet, the education sector considers this to be outside its responsibility. As time is already stretched within school curricula, additional hygiene activities are not prioritized. Where teachers are expected to deliver the HWWS intervention, participants noted a lack of motivation as it is an additional responsibility on top of their heavy workload. Activities such as hygiene clubs were identified as particularly resource intensive.

*I think challenges are there in terms of feasibility… when you have a school calendar which doesn't support some of the activities which you want to do…it creates a challenge where some of the activities are not prioritized. For example, it's the first term and you want to talk about school health clubs, yet schools are concentrating on athletics. So, you won't get that support.* —Man, national level

Participants emphasized the need for “low-resource” interventions (e.g., nudging) that require fewer skilled implementers, less time, and less money than resource-intensive interventions. They explained that these interventions would not only be more feasible to implement but also be easier to sustain after external implementers and funds are withdrawn. Participants also reported that shorter intervention sessions would better hold children’s attention and make attendance easier for children with competing commitments.

*I don't think we go through a menu of different approaches to decide but rather, say what gets us that outcome that we're looking at most effectively, efficiently, and often times it's, you know, the cost of it. So, a nudge is easier for example, and a routine is easier than doing hygiene promotion education sessions because that requires alignment with school, the class calendar, timetable and, and getting that slot, training people, it's a heavy lift.* —Woman, global level

Participants emphasized the need for interventions that require fewer skilled implementers, less time, and less money, making them more feasible to implement and to sustain.

In contrast, some participants also recommended high intervention frequency to reinforce the messages and behavioral adoption. One-day events (e.g., plays or parades) were thought to be ineffective because children have difficulty recalling the messages they hear after the event ends.

*The more that you're working with those children or adults, or whoever you're working with, the more influence you will have on them to be able to learn the appropriate behaviors that you're trying to get them to adapt. If you go in there, and you do like a 1-hour session, once a month, or once a week or something like that, yeah, I wouldn't expect any behavioral change on that, I think you're just checking the box.* —Man, national level

#### Unengaging Intervention Content

In addition to shorter and more frequent HWWS promotion activities, participants felt that more engaging content was required for effective HWWS promotion. Existing HWWS interventions targeting older children do not engage and motivate them sufficiently, focusing primarily on health messaging delivered via a didactic approach, which participants believed to be ineffective. Although most participants believed that health messaging is necessary to ensure older children understand why they are being asked to wash their hands, they felt that existing approaches are ineffective because they are not engaging. Further, these approaches do not create a link between HWWS and health that older children find tangible.

*Children do not want you to push things down on them… if you're going to teach them, lecture them like their parents and teachers do, they are going to be disconnected. So, you need to do it in a participatory manner, use play and let them express, don't restrict them to verbal exchange.* —Woman, local level

Participants believed that older children learn through play, so making sessions fun and interactive would better hold their interest and make messages more memorable, such as through games, role-play, puppets, and songs. Demonstrating HWWS to children and having them subsequently demonstrate HWWS to their peers for feedback was also an interactive technique recommended by participants. Multiple participants felt that the glitter game—where glitter represents germs and is passed around children’s hands before being washed off with soap and water—is an interactive and fun way to teach older children about the importance of HWWS and makes outcomes more tangible.

*Having fun is the most important thing that you need to encourage during the hygiene promotion sessions because if you only encourage messaging and information sharing, the children are going to retain it, of course, because they are a sponge that absorbs everything that you say, but there is not going to be change in the behavior.* —Woman, global level

Participants recommended interventions that used positive “feel-good” motivational drivers to increase older children’s HWWS, such as linking HWWS to doing well in school (due to fewer illness-related absences), completing higher education, and securing a high-paid, high-status job.

*If you wash your hands, you'll be topping your class, you'll be one of the model students, you will be getting higher marks and subsequently you will not be absent in your class because if you wash your hands you will be protected from the diseases, and then therefore the regular attendance into the class, and then also the cognition development.* —Man, global level

Material incentives, such as placing toys and games near handwashing facilities, were also thought to encourage older children to practice HWWS by making the behavior memorable. Incentivizing children to perform handwashing via competitions or with verbal encouragement (e.g., praise from caregivers) was also perceived to be a good approach.

Participants recommended leveraging social norms within the peer group. Older children spend much of their time with their peers, especially in humanitarian settings. Thus, participants believed older children were easily influenced by peers and responsive to peer pressure. Participants suggested using a peer-to-peer approach—creating “peer champions” in hygiene clubs (where a select number of children are trained to promote handwashing to their peers) or having a student monitor at the handwashing facility to encourage maintenance and use—to create social norms that encourage HWWS. Participants also believed installing group handwashing facilities and scheduling group HWWS sessions would promote consistent HWWS as a socially desirable behavior and encourage children to conform to this norm.

*It is the idea of creating that element, that peer pressure within the same age groups to say, kids will listen to what other kids say or they will understand if a demonstration is done by their peers. Or, if those people in this school health club act as role models, such that other children also see that “oh, we can also do the same.” Then, also, understanding that kids spend most of their time together as kids, even at school or outside the school.* —Man, national level

#### Non-enabling Physical Environments

Participants felt that the lack of an enabling physical environment often hinders the success of HWWS interventions targeting older children. They explained that, in settings where handwashing facilities with soap and water are lacking, if the provision of handwashing hardware is not part of the intervention, children cannot properly practice what they have learned from HWWS promotion.

Participants felt that the lack of an enabling physical environment often hinders HWWS interventions’ success targeting older children.

As well as providing handwashing hardware where needed, participants also felt that the hardware should be designed and positioned to act as a visual cue to HWWS. Strategically placing handwashing facilities at the entrance to the classroom was an example of using hardware to act as a visual cue. Nudges were commonly referenced as a good approach—painting footsteps leading from the toilets to handwashing points was frequently quoted. Participants also felt it was important to make facilities attractive and inclusive for older children. Suggestions included making facilities colorful, using cartoons or painting murals on or around facilities, and adding mirrors. Attractive handwashing facilities were thought to nurture children’s sense of ownership of the facility and encourage use. Making facilities child-friendly was also considered important, as children do not want to spend much time and effort on HWWS. Participants recommended ensuring taps are easy to operate and the height of facilities is adjusted so that children can easily reach them. Multiple participants also mentioned the need to make facilities inclusive of children with disabilities. The location of handwashing facilities was also deemed important. In schools, participants recommended installing facilities as close to the school building as possible so busy children can easily reach them.

*It's important a hand washing station is easy to use and easy to access. If it’s going to take a lot of time, or the children need to actually focus on hand washing, it’s not going to happen because the kids are going to be thinking about other things and they’re going to be looking for their friends and going out or going to school. So…it’s important that a hand washing station is something easy and simple for the children just to make it automatic.* —Woman, global level

### Stage 3: Delivery

Four major themes related to intervention delivery emerged: availability of skilled implementers, reaching out-of-school children, community mistrust, and lack of coordination. The first 3 themes were reflected across all participant levels, whereas lack of coordination emerged from interviews with participants at the global, regional, and national levels.

#### Availability of Skilled Implementers

Recruiting people with the necessary skills to promote HWWS to older children was reported as a common challenge. Working with older children requires a specific skill set that is difficult to master, even for existing hygiene promoters. Transitioning from working with adults to working with children was thought to be difficult for hygiene promoters because of the more informal interactions and focus on fun and participatory strategies (e.g., singing songs and playing games), which they may find uncomfortable. Many participants also reported that teachers often lack the skills to deliver hygiene messages to older children in a participatory manner. As such, building the capacity of those involved in intervention delivery was believed to be of fundamental importance.

*There is naturally resistance from adults not wanting to be like children, even though they want the job they don't want to be like children and play like children.* —Woman, local level

#### Reaching Out-of-School Children

Reaching children outside of schools or CFSs with HWWS interventions was perceived to be a challenge. Some children do not attend these institutions due to an insufficient number of spaces or because of work or household duties. Participants explained that attempts to reach these “out-of-school” children are mostly via one-off activities in the community that lack effectiveness and have low attendance because these children find them uninteresting or because of the competing demands on their time.

*You will compete with their time … they will not join at the start or become interested in any hygiene or health discussion because they have different interests now, because they are out of school. So, there's no environment that actually motivates them and, at the same time, supports them, for them to be able to participate in any hygiene or health education.* —Man, national level

Designing more HWWS interventions within the community, particularly at the household level, was recommended to reach these out-of-school children. Participants also felt that household-level interventions benefit from also reaching caregivers, who may support and encourage children to practice handwashing at home.

Designing more HWWS interventions within the community was recommended to reach out-of-school children.

#### Community Mistrust

Participants believed that caregivers often lack trust in hygiene promoters and hygiene promotion activities and frequently withhold permission for their children to attend hygiene promotion sessions. Mistrust of hygiene promotion was said to be especially high when delivered outside of existing structures like schools or CFSs and where sessions are “child-only,” i.e., caregivers were not invited to attend. Hygiene promoters also report feeling uncomfortable when approaching children outside of these structures.

*I think if you're trying to do something that doesn't kind of have that structure around it, it's a little bit more difficult and it's a little bit less acceptable perhaps from a parent’s point of view, in terms of just organizing, like, them not knowing who you are and not knowing what the activity is, etc.* —Female, global level

To overcome this challenge, participants recommended consulting the community to gain their buy-in and to secure parental consent for child participation.

*I feel organizations need to build a stronger constituent with the community. Number 1, the community are the gatekeepers. The communities will grant you access to their children, the community reinforce your messages, they grant you access to their children…* —Woman, local level

#### Lack of Coordination

Participants felt a lack of standardized tools for implementing both formative research and specific interventions within the NGO community hindered the effectiveness of HWWS interventions. They explained that even when there is evidence to support a specific intervention, replicating it without standardized tools is challenging. One participant referenced the difficulties in replicating a nudge intervention that was successful in a school in Bangladesh without a standardized tool.

*Other countries, 1 or 2 that I know of, have implemented it but not with any sort of global tools to support them, so they've gone about doing it in very different ways and with different levels, degrees of success.* —Woman, global level

Participants recommended a more coordinated approach to child-targeted HWWS promotion within and across sectors to encourage sharing evidence and tools. For school-based interventions, participants believed that coordination between the education sector and the community is needed so that they can better support HWWS interventions.

*So basically, the knowledge management part, sharing of best practices, sharing of resources in some pools, these actually are essential for you to be able to better help the schools and teachers to promote habit, and also stimulate the social environment part in a school setting.* —Man, national level

### Stage 4: Evaluation

Two major themes related to evaluation emerged from participant interviews: lack of evaluation rigor and failure to assign older children’s handwashing as a primary outcome in evaluations of hygiene interventions. Both themes emerged predominately from interviews with participants at the global, regional, and national levels.

#### Lack of Evaluation Rigor

Participants felt that there is a lack of rigorous evaluation of HWWS interventions targeting older children, resulting in limited evidence to assess impact, which could also encourage prioritization of older children’s HWWS and limited evidence to inform the design of future interventions. They were concerned that much of the evidence gathered by the NGO sector is anecdotal. For example, several participants described how photographs of children performing HWWS shared by parents and hygiene promoters were used as evidence that an intervention was working.

*I think one of the clear issues that we have is that we don't measure enough the impact of what we are doing. So that's a bit problematic. Like not measuring makes it hard to know what works and what doesn't.* —Man, regional level

Knowledge, attitudes, and practice surveys were frequently used to evaluate HWWS interventions. Participants recognized this as a flawed methodology because practices are often measured via self-reporting and a control group is usually lacking; they expressed concerns about relying on data from these surveys given their lack of rigor. Further, they explained that adherence to protocols can be a problem. For example, baseline surveys are sometimes undertaken after implementation begins, and “ethics obstacles” were blamed for hindering survey improvements, such as the addition of a control group.

*The baseline usually isn't done in the very beginning, it's done a couple months into it because of the time constraints and rolling out and doing it, and so it's not really a good indicator of behavioral change, or knowledge, or practices and things like that.* —Man, national level

#### Failure to Assign Older Children’s Handwashing as a Primary Outcome in Evaluations of Hygiene Interventions

Participants partly attributed the lack of rigorous evaluations of HWWS interventions targeting older children to older children’s handwashing behavior rarely being designated as a primary outcome in evaluations of hygiene interventions in general. Where handwashing behavior is measured and reported, it was said to be usually that of caregivers rather than children. Evaluations were also said to be overlooked because of the difficulties associated with measuring handwashing, particularly by observation. Where an evaluation is undertaken, knowledge of HWWS, rather than handwashing practice, is typically the outcome measured.

Participants called for more rigorous evaluations of HWWS interventions targeting older children to be undertaken and for organizations to commit to sharing these findings across the sector to grow the evidence base and lead to better-informed intervention design.

## DISCUSSION

This study offers insight into the perspectives of NGO practitioners on the challenges to HWWS interventions targeting older children and related solutions to improve effectiveness. Many of the challenges practitioners identified align with those reported in assessments of hygiene promotion interventions in school-based settings in low- and middle-income countries. These assessments found that schools often lack an enabling physical environment—functional handwashing facilities and consistent access to soap and water.^46^^–^^51^ They also report that inadequate funding, time, technical capacity, and competing classroom priorities impede the feasibility, acceptability, and adherence of hygiene promotion interventions, and, like practitioners, they call for coordination within and between sectors.

The challenges identified also align with broader social-ecological perspectives on health and health behavior and highlight the need to consider not only older children’s handwashing behavior within a multilevel context but also the programs designed to address this behavior. For example, the Integrated Behavioral Model for WASH Framework categorizes WASH determinants across 3 domains: psychosocial, technological, and contextual factors, which all operate across multiple levels of influence (individual, interpersonal, communal, and social).^52^ The challenges reported in the design and delivery of HWWS interventions targeting older children span all 3 of these conceptual domains and levels of influence. For example, engaging content and delivery impact individual-level psychosocial determinants among children. Interventions are often delivered poorly due to WASH implementers’ limited capacity to foster supportive interpersonal relationships to which older children respond. Staff retention and training are limited by larger contextual factors like short funding periods and lack of coordination across the sector. HWWS programs will not achieve the desired behavioral outcome in the absence of robust hardware, which, in turn, can be used to trigger individual-level determinants. Applying the lens of multilevel theories to the funding, design, delivery, and evaluation of HWWS programs will help further explore how the challenges described are interrelated and mutually reinforcing.

The challenges identified by practitioners highlight the need to consider not only older children’s handwashing behavior within a multilevel context but also the programs designed to address this behavior.

Despite the alignment we find between perceived challenges and theoretical behavior models, such as the one previously noted, none of the practitioners explicitly referred to or suggested using theoretically informed approaches to guide intervention design or delivery. These interventions are thought to lead to better outcomes^53^^–^^57^; however, practitioners’ failure to connect theory and practice was evident. Although practitioners emphasized the need for low-resource interventions, they also recommended various approaches (e.g., interventions using motivational drivers, leveraging social norms, and implementing nudges) without considering the associated resource burden. This suggests practitioners struggle to apply their contextual knowledge to intervention design or selection. Practitioners may benefit from using theoretical models to guide intervention design. By systematically linking HWWS determinants to specific intervention approaches, they may better ensure that interventions are contextually appropriate and address the most salient determinants in their operational context, ensuring efficient allocation of resources. However, many theoretical models are not operationally feasible, which is likely the reason for their low uptake by practitioners.^58^ They typically address barriers and enablers but do not provide guidance on selecting relevant approaches to influence them.

Theoretical models of behavior recognize the importance of individual characteristics and their broader social relationship systems. Gender, for example, has implications for expectations and norms around behaviors such as handwashing.^59^^–^^62^ However, practitioners only touched on social inclusion within the context of infrastructure, and this was largely limited to recommending that facilities were accessible to children with disabilities. None of the participants mentioned gender, and broader engagement on inclusion was largely absent. When designing and implementing HWWS programs for older children, it is important to apply a gender and social inclusion lens not only to the infrastructure component but also to the whole program. It is also important that practitioners consider gender implications when designing messages and deciding how, where, and when interventions are delivered and evaluated.

Practitioners recommended using low-resource interventions and having high intervention frequency. Resource-intensive interventions, even when effective on a small scale, present a challenge for wide-scale implementation. For example, the motivation-based SuperAmma intervention achieved a large increase in HWWS in a trial in rural Indian households,^63^ but the intervention’s scalability is limited due to its high demand on resources.^64^ Low-resource interventions are more feasible to implement at scale, and evidence suggests they can be as effective as resource-intensive interventions.^65^ Evidence also indicates that high intervention frequency is also important.^64^^,^^66^^–^^69^ Although these recommendations seem contradictory, the potential for low-dose, high-frequency interventions has been explored in various aspects of public health programming and may be a potential innovation to bring to HWWS promotion generally and to HWWS interventions targeting older children specifically.^70^^–^^73^

Resource-intensive interventions, even when effective on a small scale, present a challenge for wide-scale implementation.

Although evidence suggests that a combination of hardware and software is necessary for behavior change,^59^^,^^64^^,^^74^ in resource-scarce settings, it may prove more challenging to meet both of the previously mentioned recommendations with software-heavy interventions, which rely on human resources. Interventions that involve repeated exposure to low-resource hardware without requiring many human resources could more feasibly meet both recommendations. To achieve this, practitioners recommended using environmental nudges, small changes to the environment that cue and trigger HWWS. Environmental nudges that have been shown to increase children’s HWWS include painting brightly colored footprints leading children from the toilet to handwashing facilities,^75^^,^^76^ placing toys inside of transparent soap to incentivize soap use,^77^ and tying soap onto a piece of rope that acts as a hall pass and reminds children to wash their hands with soap after visiting the toilet.^78^ Another example of environmental nudges is strategically designing and positioning handwashing facilities to cue behavior, such as making facilities attractive, positioning facilities directly in a child’s path, or ensuring they are highly visible to heighten perceptions of social pressure to perform HWWS. Not every nudge will be contextually appropriate, so practitioners should apply contextual knowledge when designing nudge-based interventions. Nudging HWWS is only appropriate within a physically enabling environment and where children understand how to perform HWWS. In the absence of this, the intervention design would need to include other techniques, such as the provision of handwashing hardware and demonstrations of HWWS technique.

There is also evidence to support the use of social norm–based interventions using motivational drivers. Social norms have been found to be a strong determinant of handwashing behavior, including among children.^62^^,^^79^^–^^82^ A study in Bangladesh reported that children were more likely to practice HWWS after visiting the toilet when a peer monitor was present,^83^ and a study in Kenya that found hand cleaning rates were higher when at least 1 other person was present at the handwashing station.^84^ The evidence on using motivational drivers in HWWS interventions mostly results from interventions targeting adults.^63^^,^^64^^,^^66^^,^^85^ However, recent evidence suggests this approach can also increase children’s HWWS.^36^^,^^77^^,^^86^ Interventions should target the drivers relevant to children, such as “play,” “curiosity,” and “nurture.”^36^^,^^77^

Practitioners believed that existing approaches to health messaging lack effectiveness, concurring with various studies reporting health as a poor motivator of HWWS.^87^^–^^90^ However, whereas most of these studies dismissed this approach in favor of alternatives, practitioners felt that including health messaging is still important if designed to create a tangible link between HWWS and health. Recent evidence supports including health messaging and health education in HWWS interventions targeting older children. A systematic review found that providing “information about health consequences” contributes to a positive change in children’s hand hygiene behavior^36^; other studies found knowledge to be a necessary precursor for HWWS among children.^36^^,^^91^

Globally, 59 million primary school-age children are out of school.^92^ Because the overwhelming majority of HWWS interventions targeting older children have been implemented in schools,^11^^,^^34^^,^^93^ it is evident that this vulnerable group has been historically overlooked. Though schools are an important setting to implement HWWS interventions for children, community-based interventions, including at the household level, are necessary to reach out-of-school children. Household-level delivery may also help to ease the mistrust by allowing caregivers to oversee activities, which may encourage caregivers to support children in sustaining new handwashing habits. Since a likely deterrent to organizations adopting household-level HWWS interventions is the concern that they are more resource intensive than school-based interventions, the need for low-resource interventions is even more apparent.

Respondents’ call to build the evidence base around HWWS promotion among older children is well founded. Multiple systematic reviews of handwashing interventions reveal that most published studies focus on adult-targeted interventions and are not of high quality.^11^^,^^34^^,^^35^^,^^59^^,^^93^ This hinders the ability to draw conclusions about the best approaches for targeting older children. A study of humanitarian perspectives on the Ebola WASH response in Liberia reported that organizations mostly focused monitoring on inputs and outputs, and none systematically monitored outcomes related to hygiene knowledge, awareness, or behavior.^38^ Understandably, practitioners were concerned that older children are not prioritized for HWWS interventions, hampering efforts to improve their HWWS behavior. Building the evidence base will hopefully demonstrate the value of improving older children’s HWWS behavior, influence policies and standards, and encourage donors and NGOs to designate more funding and resources for HWWS interventions targeting older children. However, practitioners must also engage with the existing evidence base, given there have been some rigorous trials of school-based HWWS interventions. Some of the responses given by practitioners in this study suggest this is not always the case. Group handwashing, for example, was often proposed as an effective approach yet evidence suggests it is unlikely to achieve behavior change, especially outside of a larger behavior change intervention package.^69^^,^^94^^,^^95^ The gap between research and practice is a long-standing problem and calls for better ways of disseminating the evidence to make it accessible to practitioners.^96^ Rather than relying only on scientific publications, additional channels are needed to reach practitioners, for example, via policy briefs, one-on-one meetings, workshops, and seminars.

Building the evidence base will hopefully demonstrate the value of improving older children’s HWWS behavior, influence policies and standards, and encourage donors and NGOs to designate more funding and resources for HWWS interventions targeting older children.

Finally, participants called for better coordination within the NGO community to encourage sharing of best practices and the development and access to standardized tools that aid the implementation of formative research and of interventions that are simple, rapid to employ, and reflective of the local context. The need for a well-coordinated approach to achieve effective and sustainable HWWS interventions has also been identified by others^37^^,^^97^ and extends beyond just coordination within the NGO community. Organizations should foster intersectoral collaboration and coordination. In addition, they should create stronger links with (1) national governments to understand their policies on hygiene and help formulate new contextually appropriate strategies, (2) donor agencies to secure sufficient and dedicated funds, (3) educational institutions to firmly integrate HWWS promotion within the curriculum, and (4) academia to support rigorous evaluation and dissemination of information from the organization to policymakers and other key actors. To achieve long-term impact, it is also necessary to engage the community to plan how the intervention will be sustained beyond the withdrawal of the implementing organization.

To achieve long-term impact, it is necessary to engage the community to plan how the intervention will be sustained beyond the withdrawal of the implementing organization.

As school is the most common setting for HWWS interventions targeting children, engaging with educational institutions is particularly important. The NGO sector plays a key role in supporting the education sector to make institutional-level changes that establish HWWS promotion as a key part of the curriculum and implement systems that support its delivery. For HWWS interventions to be successful and sustainable in schools, the buy-in and cooperation of schoolteachers and school managers are essential. Programs should encourage teachers and managers, as duty bearers with responsibility for children’s health, to view handwashing as a life skill that children need to improve their overall health and should work toward making handwashing promotion a core subject within national teacher training programs.^98^

Schools themselves are a part of a complex system, and thus any school-based intervention relies on intersectoral collaboration. Conducive government policies, community support, and school action are all needed for successful interventions to be sustained. For example, cooperation between ministries of education and other ministries, such as health, public works, finance, local governance, and water authorities, may be required for school-based HWWS interventions to be successfully sustained.^98^ Gaining political support and commitment is important to ensure WASH in schools, which includes HWWS interventions, is upheld as an essential part of an education program and to ensure allocation of sufficient financial and human resources to support sustainability. NGOs or donor agencies may provide the investment costs, but for a school-based HWWS intervention to have long-term sustainability, governments, schools, and communities should ultimately cover operation, maintenance, and replacement costs of educational materials, facilities, and supplies.^98^ National financial policies on WASH in schools that support these costs are essential. Involving parents and the community in school-based HWWS programs fosters a sense of ownership, especially important when their support is needed through financial donations, unskilled labor, and the provision of local construction materials to build and maintain handwashing facilities. Parental involvement is also important to support children to apply what they learned in schools at home. Involvement may be in the form of school management committees, parent-teacher associations, or committees specifically set up for WASH in schools.^99^

### Limitations

There are some important limitations of our study. First, given that both the researchers and the participants work in the field of WASH, there were instances where participants already knew the researchers and their work, and the lead researcher had prior professional relationships with 3 participants. This may have biased data collection and analysis, as well as influenced participants’ responses, subjecting our findings to social desirability bias. Second, although most participants spoke openly, it seemed that some were hesitant to express personal views outside of their organization’s official stance. We tried to minimize this by emphasizing to participants at the start of the study that we were interested in both positive and negative views and by ensuring only nonleading questions were posed. Nonetheless, some responses may not have been truly representative of the participants’ personal views. Thirdly, due to the qualitative nature of our study, findings cannot be generalized to all NGO practitioners or all NGOs.

## CONCLUSIONS

This study identifies challenges in designing and implementing effective HWWS interventions targeting older children and solutions to these challenges from the NGO practitioner perspective. Practitioners believe the NGO sector should make children’s handwashing a top priority. Practitioners strongly advocate for better intra- and intersectoral coordination to overcome challenges related to the integration of HWWS promotion within existing educational institutions, the standardization of implementation tools, and the sustainability of interventions. We also recommend that practitioners engage more with theory when designing interventions to support application of their contextual knowledge to intervention design.

## Supplementary Material

GHSP-D-22-00231-Supplements.pdf

## Notes

**Peer Reviewed**

**First published online:** January 18, 2023.
