# Supplementary material for: Nongovernmental Organization Practitioners’ Perspectives on the Challenges and Solutions to Changing Handwashing Behavior in Older Children: A Qualitative Study
Source: Glob Health Sci Pract. 2023 Feb 28;11(1):e2200231. doi: 10.9745/GHSP-D-22-00231 (PMC9972390; doi:10.9745/GHSP-D-22-00231)
Supplement: GHSP-D-22-00231-Supplements.pdf [file GHSP-D-22-00231-Supplements.pdf]

Supplement to: Watson J, Cumming O, Dreibelbis R. NGO practitioner's perspectives on the challenges and solutions to changing handwashing behavior in older children: a qualitative study. *Glob Health Sci Pract*. 2023;11(1):e2200231. https://doi.org/10.9745/GHSP-D-22-00231

Supplement 1. Semi-Structured Interview Guide

Introduction

Thank you for agreeing to take part in this interview. My name is XX and I am a researcher at the London School of Hygiene and Tropical Medicine. I am conducting a piece of research exploring organisational approaches to handwashing promotion in emergency and development responses and trying to understand the factors behind the decision-making process.

Just to clarify so that we are on the same page, since there can be many different interpretations of the term handwashing promotion, here what I mean by handwashing promotion is any activity designed to increase handwashing with soap. So that includes messaging and education as well as infrastructural improvements such as providing soap and handwashing stations.

[The following questions serve as a guide for discussion but can be flexible so long as the topic of conversation remains on the broader context of handwashing promotion for children. Probes are in bullet points]

Background Information

1. Could you briefly describe your current role and how you came to work in the WASH sector and this organisation?

    - Organisation and role
    - Length of time in role
    - Country of work
    - Previous experiences
    - Particular interests

2. In what ways has your organisation been involved with commissioning or implementing handwashing promotion for children?

Understanding of determinants

Firstly, before we delve more specifically into the programmes you have implemented, I'd like to ask you to think about children's handwashing more broadly. I'm going to share my screen with you – this is a broad way we think the determinants of handwashing could be categorised.

3. Looking at these, which do you think are the most important determinants of child handwashing?

    - Do you think this is any different in emergency settings/outbreak contexts?

Intervention selection and design

Now, I will move more specifically to your organisation's approaches.

4. Who is involved in the decision-making around which handwashing promotion programmes for children are implemented by your organisation?

    - How much input does the country office have?
    - Who makes the final decision?

5. If presented with a selection of handwashing promotion interventions or programmes your organisation could implement, what factors do you consider when choosing which one to implement?

    - Is a needs assessment done?
    - Is any formative research conducted in order to determine the barriers and enablers to handwashing?
    - Do you consider what motivates children, hardware available, cultural factors?
    - Dependent on funding available?
    - Dictated by what funders wants?
    - Guided by published research?
    - Availability of resources?

Handwashing Promotion Approaches

6. Can you tell me about a few specific child-focused handwashing-promotion interventions your organisation has funded or implemented?

    For each intervention probe on:

    - Where was this? Location and context
    - What age groups were targeted?
    - What did this intervention involve: what hardware, software, specific activities?
    - What were the key messages and what channels were used for message delivery?
    - What was the mechanism of action – how does this intervention lead to a change in behaviour?
    - Who implemented the intervention?
    - What was your organisation's role – implementer/funder?
    - How long was the intervention and how much was it repeated?
    - What was the time scale for delivery?

7. Do you think this/these interventions appropriately address what you feel are the determinants of handwashing behaviour and did they motivate children?

    - Why/why not?
    - What are the barriers?
    - What should have been different?

Effectiveness

8. Can you tell me about the most successful handwashing promotion program that you have worked on for children?
    - Probe on details of the program:
        - Where,
        - Age group
        - Activities
9. What determinants do you think this program focussed on?

10. What do you think led to the success?
    - What components were particularly effective?
    - What makes these components effective (probe further for what determines child handwashing behaviour)?
    - What components were less effective, why?
    - Any other examples (follow the same probes)?

11. How did you know this program was effective?
    - What evaluation was done?

12. Are there any examples of less successful programmes you have worked on?
    - Why did they not work?
    - Probe on approach, funding, delivery

Acceptability and Feasibility

13. What do you think determines the acceptability of behaviour change interventions for children in these settings?
    - Cultural norms, settings, perception, delivery

14. How have the approaches you have talked about been perceived among the community?
    - Which approaches have been the most accepted among the community and why?

15. How have they been perceived among the children?
    - What approaches have been the most popular among children and why?

16. Thinking about the different approaches you have taken, how feasible/practical have these been to implement in the field?
    - Were they well understood by field staff and did they find it easy to implement as directed?
    - Were there any time constraints or anything that made it difficult to implement as directed?
    - Were they able to reach the right audience?
    - Were they easy to repeat?

Challenges

17. What are the challenges facing programs targeted at promoting children's handwashing?
    - How are these challenges different to those facing adult-focused programs?

18. Are there any challenges specific to promoting handwashing to children in emergency settings/responses?

Future Recommendations

19. Given your knowledge and experience, what are your recommendations for future handwashing promotion interventions targeted at children in an emergency setting or during an emergency response such as the COVID-19 response?

Ending question

20. Are there any last comments or questions before we end the interview?

Supplement to: Watson J, Cumming O, Dreibelbis R. NGO practitioner's perspectives on the challenges and solutions to changing handwashing behavior in older children: a qualitative study. *Glob Health Sci Pract*. 2023;11(1):e2200231. https://doi.org/10.9745/GHSP-D-22-00231

Supplement 2. Thematic Analysis: Coding Structure

Programme Cycle Stage	Themes	Codes
**Funding Acquisition**	Lack of prioritisation	Handwashing promotion not prioritised
		Children not prioritised
		Focus on under-fives
		Personal interests dictate programme focus
		Insufficient funding
	Funding inconsistency	Short-term funding
		Changing programme leads
**Design**	Insufficient formative research	No formative research
		Inadequate formative research
		Consider context
		Consult children
		Tailor intervention
	Demand on resources	Resource-intensive approaches
		Difficulty accessing teaching materials
		Difficulty maintaining facilities
		Limited physical space
		Limited time
		Difficult to integrated into curricula
		Teachers' motivation low
		Sustainability issues
		Low-resource interventions needed
		High intervention frequency needed
		Sustainable interventions needed
	Unengaging intervention content	Children lack motivation to participate
		Reliance on didactic approach
		Health messages unengaging
		Health knowledge important but not sufficient
		Motivational drivers
		Incentives
		Competitions
		Leveraging social norms
		Demonstrations
		Interactive, fun, engaging approaches
	Non-enabling physical environments	Lack of handwashing facilities and materials
		Provide hardware
		Make facilities attractive
		Make facilities child-friendly
		Use environmental cues
**Delivery**	Availability of skilled implementers	Lack of skilled staff
		Hygiene promoter unwillingness
		Building capacity
		Hire people with right skills
	Reaching out-of-school children	Difficult reaching children outside of schools and CFS
		Delivery mainly in schools and hygiene clubs
		Increase delivery in community
	Community mistrust	Mistrust within community
		Engage community
	Lack of coordination	Lack of standardised tools
		Difficulty standardising tools

Supplement to: Watson J, Cumming O, Dreibelbis R. NGO practitioner's perspectives on the challenges and solutions to changing handwashing behavior in older children: a qualitative study. *Glob Health Sci Pract*. 2023;11(1):e2200231. https://doi.org/10.9745/GHSP-D-22-00231

		Complicated tools
		Need for coordination
**Evaluation**	Lack of evaluation rigour	Lack of evaluation
		Anecdotal evidence
		Poor adherence to protocol
		Difficulty measuring handwashing
		Reliance on KAP surveys
		Rigorous evaluations needed
	Failure to assign older children handwashing as a primary outcome in evaluations of hygiene interventions	Handwashing not a major outcome
		Knowledge measured over behaviour
